# Disentangling the effects of metabolic cost and accuracy on movement speed

**DOI:** 10.1371/journal.pcbi.1012169

**Published:** 2024-05-31

**Authors:** Garrick W. Bruening, Robert J. Courter, Shruthi Sukumar, Megan K. O’Brien, Alaa A. Ahmed

**Affiliations:** 1 Department of Integrative Physiology, University of Colorado, Boulder, Colorado, United States of America; 2 Department of Computer Science, University of Colorado, Boulder, Colorado, United States of America; 3 Shirley Ryan Ability Lab, Northwestern University, Chicago, Illinois, United States of America; 4 Department of Mechanical Engineering, University of Colorado, Boulder, Colorado, United States of America; Johns Hopkins University, UNITED STATES

## Abstract

On any given day, we make countless reaching movements to objects around us. While such ubiquity may suggest uniformity, each movement’s speed is unique—why is this? Reach speed is known to be influenced by accuracy; we slow down to sustain high accuracy. However, in other forms of movement like walking or running, metabolic cost is often the primary determinant of movement speed. Here we bridge this gap and ask: how do metabolic cost and accuracy interact to determine speed of reaching movements? First, we systematically measure the effect of increasing mass on the metabolic cost of reaching across a range of movement speeds. Next, in a sequence of three experiments, we examine how added mass affects preferred reaching speed across changing accuracy requirements. We find that, while added mass consistently increases metabolic cost thereby leading to slower metabolically optimal movement speeds, self-selected reach speeds are slower than those predicted by an optimization of metabolic cost alone. We then demonstrate how a single model that considers both accuracy and metabolic costs can explain preferred movement speeds. Together, our findings provide a unifying framework to illuminate the combined effects of metabolic cost and accuracy on movement speed and highlight the integral role metabolic cost plays in determining reach speed.

## Author summary

How the brain implicitly controls movement speed remains unclear despite the prevalence of anomalous movement slowing in a range of movement and mental health disorders. Reaching speed has long been thought to be dictated by accuracy. Despite the predominant influence of metabolic cost on walking speed, any influence of effort on the control of speed in relatively low effort reaching movements has been underappreciated. Here we show that the implicit control of reaching speed is influenced by both the metabolic and accuracy requirements of the movement. We next propose a mathematical model that explains how accuracy, metabolic expenditure, and time costs, interact to determine the implicit choice of movement speed. Our results highlight that disease-related movement slowing can be modulated by factors beyond accuracy. Importantly, we show that movement costs such as metabolic cost have a strong influence, despite the relatively low cost of reaching. Economic variables such as time and subjective effort costs can also lead to movement slowing. Taken together, we present a unifying framework with which to interrogate the critical role accuracy, effort and time costs play in the implicit control of movement speed in health and disease.

## Introduction

A defining characteristic of any movement is the speed, or *vigor*, with which it is performed. However, the speed of our movements is not a stereotyped quantity; rather, it reflects the underlying value associated with performing the movement [1,2]. There is a wealth of data demonstrating that animals and humans move faster towards more rewarding targets [3–6], as well as to the same target but in more rewarding environments [7,8]. For example, in humans, rewarded targets elicit higher reach speed even when these rewards are abstract and noncontingent on performance [9]. Selection of a movement and its speed is thus a tangible correlate of the neural representation of its utility.

Whether or not they are explicitly rewarding, all movements have something in common, they incur a primary objective cost is typically represented as metabolic energy expenditure [10]. For instance, in locomotion, metabolic energy minimization explains the selection of speed, step frequency and stride length [10–12]. In fact, these kinematics are often selected in a manner that minimize effort when represented as metabolic cost per unit distance, thus metabolic cost is considered a driving force in determining the speed of locomotion.

The role of metabolic cost in determining reaching speed, however, remains undetermined. Recent evidence suggests that that the metabolic rate of reaching movements changes with relative smoothness [13] and speed [14], and that reaching movements are likewise sensitive to effort costs [15]. For example, we reach slower in directions that involve moving more mass and prefer to reach in directions of lower effort [16,17]. While these data directly implicate effort costs in the selection of reach speed, this effort-dependence has not been confirmed through collection of metabolic expenditure data.

Rather, in goal-directed movements like reaching, accuracy has been considered the dominant cost. Faster and quicker movements tend to be less accurate, known as the speed-accuracy tradeoff, leading to slower speed selection in the face of high accuracy demands [18]. Accordingly, decreased reach speed is often attributed to increased accuracy constraints associated with the target, whereby individuals will opt to slow their movements to ensure they accurately reach the goal [19].

Therefore, distinct costs and goals of a given movement–effort, accuracy, reward, and time, among others–interact and influence the chosen movement speed. Even in locomotion, results suggest that factors other than cost per unit distance explain speed. For example, the metabolic cost of walking increases with heavier carried loads but does not affect the metabolically optimal speed [20]. Despite this, humans walk slower with greater loads [21,22], suggesting that minimizing metabolic cost per distance is not the sole factor determining preferred locomotion speed. Walking also incurs a cost of time. Preferred walking speeds vary with distance, with speeds at shorter distances dominated by a desire to save time rather than energy [22]. Individuals even prefer to run than walk, saving time at the expense of a higher cost per unit distance [23]. Unlike walking, however, reaching carries a distinct cost of accuracy. To successfully complete a movement, we need to be able to accurately reach the "reward". Higher value rewards will invigorate reaching movements while increased accuracy requirements will slow the arm. On the other hand, moving faster incurs heightened metabolic costs. While there have been notable efforts in the past to understand how accuracy and effort costs are traded off to determine reaching speed using optimal control [24–26], the interplay between accuracy and metabolic costs, in particular, in determining reach speed remain undetermined.

The primary aim of this work is to provide a comprehensive examination into the interacting roles of effort and accuracy in control of reach speed. To this end, we first measured metabolic costs of reaching movements, as a representation of effort, and demonstrated that gross metabolic costs increase with both speed and the mass required to transport at the hand. Secondly, we quantified preferred reaching speeds in an array of loading conditions and accuracy constraints and established that self-selected speeds become slower when effort costs increase, even when controlling for accuracy [18]. Next, we used a neuroeconomic modeling approach to determine the combined influence of gross metabolic and accuracy costs on the selection of movement speed. We found that increasing the gross metabolic cost of reaching by manipulating added mass at the arm significantly decreased self-selected reaching speeds. Additionally, we found that maximization of net reward rate, which considered this gross metabolic cost as well as the cost of making inaccurate movements, provided excellent predictions of preferred movement speed. While minimizing metabolic cost alone could not fully capture preferred movement speeds, these results highlight that metabolic energy unequivocally influences the selection of preferred reaching speed, though it was not the sole determinant. Rather, combining effort with accuracy and time we provide evidence for a neuroeconomic framework based on net reward rate that determines movement speed.

## Results

Our first step towards understanding the effect of effort on preferred movement speed was to quantify it. To do so, we considered gross metabolic cost as an objective representation of effort and measured the costs of reaching while changing speed and mass at the hand.

### The effect of mass on the metabolic cost of reaching

**Fig 1:**
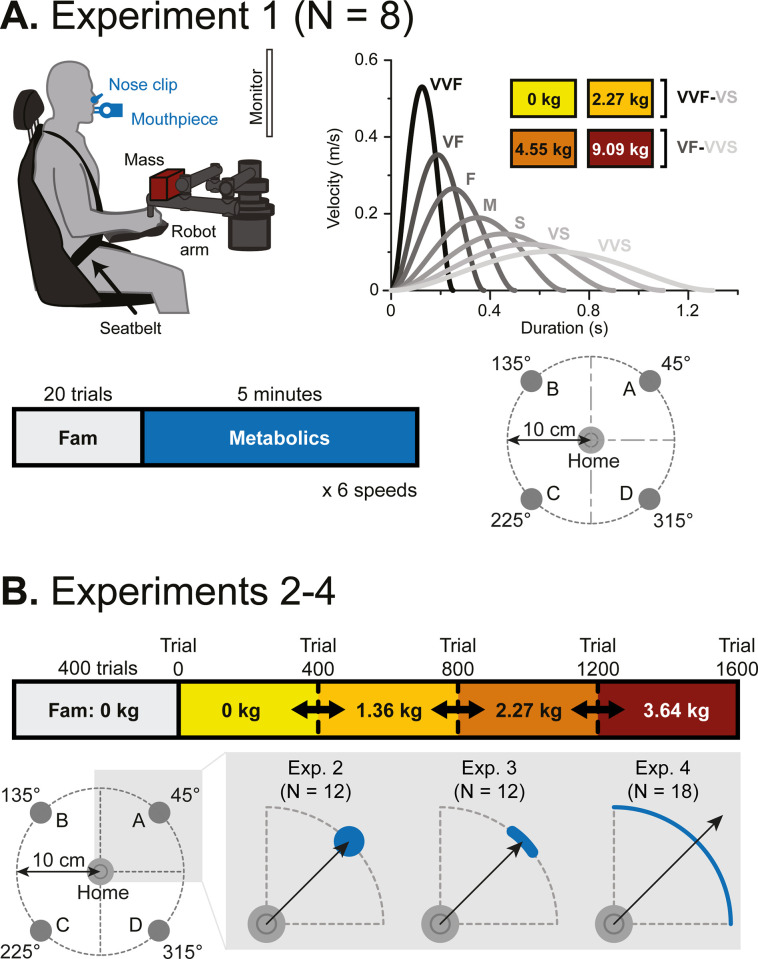
Experimental setup. (A) Experiment 1. *Top Left*. Subjects made horizontal planar reaching movements while breathing into a mouthpiece. Mass was added at the hand. *Top right*. There were seven distinct speeds, and subjects completed six of those speeds within each mass. The two heaviest masses corresponded with the six slowest speeds: Very Fast (VF, 0.325–0.425 s) to Very, Very Slow (VVS, 1.25–1.35 s). The two lighter masses corresponded with the six faster speeds: Very, Very Fast (VVF, 0.225–0.275 s) to Very Slow (VS, 1.05–1.15). *Bottom right*. Subjects made out-then-back reaching movements across a range of added masses and speeds to four targets (r = 1.4 cm) 10 cm from the home circle. *Bottom left*. The number of trials within each speed was set to allow for approximately five minutes of reaching. (B) Experiments 2–4. *Top row*. Subjects underwent five blocks of reaching movements including a familiarization block and four added mass blocks. The order of mass conditions was randomized for each subject. The general setup is the same as experiment 1. Subjects completed 400 trials in familiarization and each mass condition. *Bottom row*. Subjects made goal-directed reaching movements from a home circle and stopped at one of four targets, 10 cm away from the home circle. In experiment 2, subjects needed to stop in a circular target of the same size as experiment 1 (r = 1.4 cm). In experiment 3, subjects needed to stop in a smaller, thin, arc-shaped target (7 degrees with 1 cm thickness). In experiment 4, subjects did not need to stop and performed out-and-back reaching movements, with the only accuracy criteria that they cross the perimeter of the outer circle within the 90 degree quadrant.

Healthy, young participants (N = 8) made 10 cm reaching movements to move a cursor (r = 0.4 cm) toward circular targets (r = 1.4 cm) at six prescribed speeds (ranging from Very, Very Slow [VVS, 1.25–1.35 s] to Very, Very Fast [VVF, 0.225–0.275 s]) with four different masses (0 kg, 2.3 kg, 4.5 kg, and 9.1 kg) added at the hand for a total of 24 sets of reaching conditions (Fig 1A). Conditions were blocked such that each consisted of five minutes of reaching (~200 trials). As they performed the task, we measured metabolic rate via expired gas analysis. Metabolic rate was calculated at steady state from the final three minutes of reaching within each block.

#### Metabolic expenditure increases with added mass under varying speed conditions

**Fig 2:**
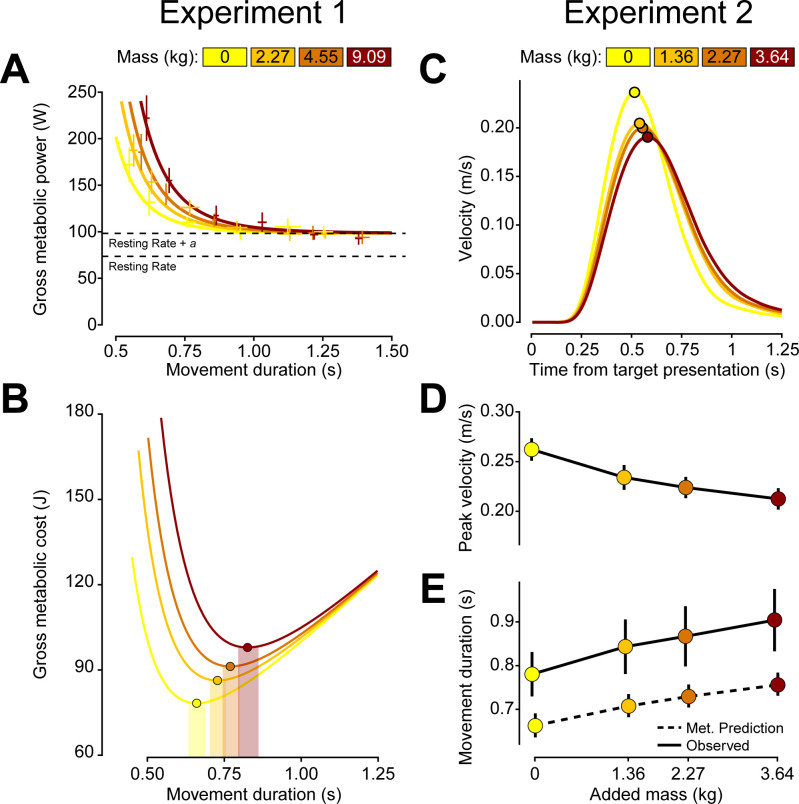
*Left column*. Experiment 1 results. (A) Gross metabolic power increases with added mass and movement speed (shorter durations). Error bars are ± standard error of the observed data. (B) Gross metabolic cost shows a distinct minimum, indicated by a circle, and this minimum duration increases with added mass. Shaded regions are the bootstrapped 95% confidence interval of the speeds which minimize gross metabolic cost. *Right column*. Experiment 2 results. (C) Average velocity traces per each added mass condition. Each line represents the subject average for one mass condition, with the peak velocity indicated by a circle. In panels D and E, each point represents the subject average for a specific mass condition. (D) Mass reduced observed peak velocity. (E) Mass increased observed movement duration. The dashed line indicates the predictions made from minimizing gross metabolic cost. Error bars are 95% confidence intervals.

Before participants performed the reaching task, we measured their resting metabolic rate, $\dot{e_{r}}$ in three, five-minute baseline periods as they sat quietly in the experimental chair. On average, the resting metabolic power was $\dot{e_{r}}$ = 73.33 ± 3.60 W (all metrics and parameter fits are reported as mean ± standard error, unless stated otherwise). As they performed the reaching task, log-transformed gross metabolic power increased with log-transformed movement speed (*β* = -0.767 ± 0.038, p < 2e-16) (Fig 2A); with no added mass, gross metabolic power ranged from 92.78 ± 6.63 W for the slowest reach to 171.98 ± 18.51 W for the fastest reach. Furthermore, across movement speeds, log-transformed gross metabolic power increased significantly with added mass (*β* = 0.017 ± 0.003, p = 2.52e-7) (Fig 2A). For a movement at the second fastest speed condition, adding 9.1 kg of mass at the hand led to an increase in gross metabolic power from 131.65 ± 14.32 W to 222.06 ± 24.39 W, a nearly 70% increase. Thus, faster reaches and greater mass both led to increased metabolic expenditure.

We next sought to determine how mass influenced the metabolically optimal movement speed, i.e., the speed at which the gross metabolic cost of the movement was at a minimum. We focus on gross cost rather than net, as gross cost minimization not only is more representative of preferred walking speeds but has also been suggested to be more appropriate given the foraging-type task studied here [22,27]. To determine the metabolically optimal movement duration, we parameterized gross metabolic rate as a function of mass and movement duration. We fit the metabolic rate data to an equation of the following form based upon the observed effects of mass on the metabolic rate of walking [15]:

$${\dot{e}}_{m}=a+\frac{bm^{i}}{t_{m}^{j}}$$ (1)

In Eq 1, *m* represents the effective mass of the arm with the added mass (see Methods), and *t*_*m*_ is the movement duration. The best fit parameters were *a* = 98.25 ± 3.05, *b* = 0.86 ± 0.43, *i* = 0.83 ± 0.10, and *j* = 5.83 ± 0.60 (MSE = 656.38 W^2^, AIC = 1750.83).

To obtain the total metabolic cost of a reach in joules (*e*_*m*_), the metabolic rate (${\dot{e}}_{m}$) measured in (W or Joules/s) is multiplied by the average movement duration *t*_*m*_, giving us gross metabolic cost:

$$e_{m}=at_{m}+\frac{bm^{i}}{t_{m}^{j-1}}$$ (2)

Indeed, the empirical data revealed that gross metabolic costs of the fastest reaches were high, reducing as the movement slows down, but then increasing again at slower speeds (Fig 2B). The minima of these curves represent the movement duration that minimizes the gross metabolic cost of the reach (Table 1). We see that not only does the metabolic minimum shift to longer movement durations at higher added load conditions, but the metabolic minimum cost increases with added mass.

**Table 1:** Experiment 1 predicted movement durations which minimize the gross metabolic cost at a given added mass. Mean [95% CI] determined from 1000 bootstrap replicates.

Mass (kg)	Metabolic Prediction (s)	Minimum Cost (J)
0	0.660 [0.634, 0.688]	78.239 [74.943, 81.862]
2.27	0.728 [0.703, 0.755]	86.245 [82.788, 90.172]
4.55	0.769 [0.745, 0.799]	91.182 [87.264, 95.486]
9.09	0.826 [0.795, 0.861]	97.865 [93.275, 103.172]

Therefore, the data from experiment 1 reveal that it is more energetically costly to make reaching movements with greater mass and that the metabolically optimal movement duration increases with added mass. In other words, gross metabolic energy minimization predicts slower movements with added mass.

### Added mass led to longer self-selected movement durations

Given that added mass leads to an increase in the gross metabolic cost of reaching, and that the metabolically optimal duration increases with added mass, we asked how this increased effort cost would influence an individual’s preferred movement speed. Will people choose to reach at the metabolically optimal speed? A second experiment was therefore designed to answer this question. Similar to experiment 1, participants made goal-directed reaching movements with added mass at the hand (Fig 1B). However, instead of reaching at a prescribed speed, participants were free to reach at a self-selected speed.

At the beginning of each trial, one of four circular targets (r = 1.4 cm) would appear centered on the circumference of a 10 cm circle and participants were asked to reach and stop the cursor (r = 0.4 cm) in the target to complete the trial. The experiment consisted of 1600 total trials, divided into four sets of 400 trials each. In each set of 400 trials, a different mass was added to the robot handle. The amount of added mass was hidden from the participant using an opaque container and could be 0 kg, 1.36 kg, 2.27 kg or 3.64 kg (Fig 1B). All participants experienced each mass condition once, and the order was randomized across participants.

Added mass led participants to make significantly slower movements as evidenced by significantly longer movement durations (*β* = 0.032 ± 0.001, p < 2e-16) and lower peak velocities (*β* = -0.013 ± 0.0002, p < 2e-16) (Fig 2C–2E). All else equal, when added mass at the arm was increased, individuals consistently opted for a slower movement speed. Therefore, not only do changes in added mass result in modulation of the energetics associated with the movement, but these changes in metabolic cost appear to influence the selection of reach speed.

### Predicting preferred movement duration

In experiments 1 and 2, we observed that mass increases the effort cost of movement and that subjects prefer slower movement durations for greater added mass. Can we explain these effort-based changes in movement preference in the context of a movement utility that is conserved across individuals? As a first step, we looked at what gross metabolic minimization would predict. For the masses used in experiment 2, we re-calculated the metabolically optimal durations using Eq 2 (Table 2).

**Table 2:** Predicted movement durations which minimize the gross metabolic cost using the masses from experiment 2. Mean [95% CI] determined from 1000 bootstrap replicates for the minimum cost and predicted duration. Mean [95% CI] for observed durations estimated using a t distribution with df = 11. MSE = 1.85e-02 s^2^.

Mass (kg)	Observed Duration (s)	Metabolic Prediction (s)	Minimum Cost (J)
0	0.780 [0.730, 0.831]	0.663 [0.636, 0.691]	78.528 [75.264, 82.157]
1.36	0.843 [0.781, 0.906]	0.708 [0.682, 0.735]	83.828 [80.523, 87.664]
2.27	0.867 [0.798, 0.936]	0.729 [0.704, 0.757]	86.407 [82.937, 90.322]
3.64	0.904 [0.833, 0.975]	0.756 [0.731, 0.784]	89.547 [85.789, 93.612]

Critically, the durations that minimized gross metabolic cost were significantly faster than the observed durations in experiment 2 (Table 2 and Fig 2E). Even though the predictions were off in absolute terms, it is worth noting that metabolic cost performs remarkably in predicting the degree of movement slowing due to mass. The slope of the relationship between predicted movement duration and added mass is very similar to the slope of the empirical data, suggesting that the brain is accounting for metabolic cost in the selection of movement duration. Nonetheless, while gross metabolic cost minimization correctly predicted a general slowing of movement as well as the degree of slowing, it could not fully explain preferred movement durations (Fig 2E). These data suggest that additional factors beyond effort are being accounted for when prospectively optimizing movement speed.

#### Maximization of net reward rate explains the combined effect of effort and accuracy costs on preferred movement duration

If we assume that movements are made to optimize a certain objective, how does the metabolic cost of a reaching movement modulate the objective function optimized by the movement? Other factors, like reward, time, and accuracy constraints of a movement can also affect the selected speed [9,18]. Assuming that the purpose of movement is to successfully acquire reward as quickly as possible but with minimal effort, we can define the utility of movement, *J*, as a net reward rate: the expected reward minus the associated costs of the movements, all divided by the total time taken to acquire the reward:

$$J=\frac{\alpha -E}{T}$$ (3)

where α is the reward to be obtained, *E* is the effort cost associated with obtaining the reward, and *T* is the total time spent acquiring the reward. Substituting the terms in Eq 3 specific to reaching movements, we represent the movement effort as measured gross metabolic cost of a reach with a given mass and duration, *e*_*m*_ (Eq 2). Additionally, there is also the time spent preparing the movement, i.e., the reaction time. We assumed the effort cost associated with reaction time, *e*_*r*_, is equal to the metabolic expenditure when not reaching—the resting metabolic rate; this cost is represented as ${\dot{e}}_{r}$ multiplied by the reaction time, *t*_*r*_:

$$e_{r}={\dot{e}}_{r}t_{r}$$ (4)

The total time to reward, *T*, then is the sum of the reaction time (*t*_*r*_) and movement duration (*t*_*m*_). Finally, we have the expected reward, which is given by the probability of completing the movement successfully at a given mass and movement duration, *P*(α|*t*_*m*_,*m*), multiplied by the reward α. Therefore, we obtain our final utility function for completing a movement:

$$J=\frac{\alpha P(\alpha |t_{m},m)-e_{r}-e_{m}}{t_{r}+t_{m}}$$ (5)

Critically, *P*(α|*t*_*m*_,*m*) represents the speed-accuracy tradeoff [18]; the shorter the duration of the movement, the lower the probability of accurately completing it. This probability was modelled as a logistic function of movement duration and added mass:

$$P(\alpha |t_{m},m)=\frac{1}{1+e^{-\beta_{0}-\beta_{1}t_{m}-\beta_{2}m}}$$ (6)

To obtain empirical estimates of the *β* parameters in Eq 6, we fit the endpoint accuracy data from experiment 1 to capture the relationship between movement duration, mass, and the probability of successful movement completion using a logit-linked binomial generalized linear mixed model (Eq 7). Successful movements in experiment 1 are those that end in the target of radius 1.4 cm. Any movement that ends outside is considered unsuccessful:

$$ln(\frac{P}{1-P})=\beta_{0}+\beta_{1}t_{m}+\beta_{2}m$$ (7)

**Fig 3:**
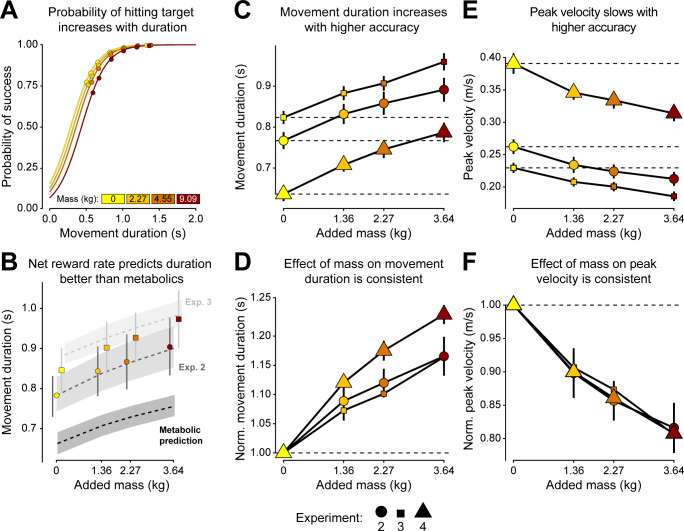
*Left column*. Modeling. (A) Function for the speed-accuracy trade off. Using the data from experiment 1, and the logistic regression shown in Eq 6, we can compute the probability of success based on target accuracy. Data points are the fraction of trials within that condition that were a success. Each line is colored by mass added from experiment 1. (B) Optimal movement durations obtained by maximizing net reward rate for experiments 2 (dark gray) and 3 (light gray), or from minimizing gross metabolic cost (black). Dashed lines and shaded regions indicate model predictions and 95% bootstrapped confidence intervals. Points are the average observed data and 95% confidence intervals, respectively, for experiments 2 (circles) and 3 (squares). *Middle and right columns*. Experiment 2, 3, and 4 results. In all panels, each point represents the subject average for a specific mass condition. Error bars show standard error across subject averages. Horizontal dashed lines show the average value for 0 kg of added mass. (C) Mass and higher accuracy constraints increased movement duration. (E) Mass and higher accuracy constraints reduced peak velocity. Panels D and F: Movement metrics normalized as a fraction of each subject’s 0 kg condition. (D) Movement duration and (F) peak velocity exhibited similar changes due to mass regardless of accuracy requirements.

Fig 3A shows the fit to the probabilities measured from the data in experiment 1 to the corresponding parameters in Eqs 6 and 7 (*β*_0_ = -1.20 ± 0.28; *β*_1_ = 5.96 –± 0.19; *β*_2_ = -0.11 ± 0.01; all p < 1.5e-05). Using these parameters for *P*(α|*t*_*m*_,*m*) and the measured reaction times from experiment 2, we then used the net reward rate model in Eq 5 to predict the observed movement durations in the data from experiment 2, fitting reward α as a free parameter. We found that net reward rate described preferred movement duration better than gross metabolic cost alone (α = 57.18 [36.67, 82.39] 95% CI; MSE = 1.43e-04 s^2^; Fig 3B). The predicted movement durations for experiment 2 are compared to observed durations in Table 3.

**Table 3:** Experiment 2 observed durations compared to those predicted by maximizing net reward rate. The fitted free parameter, α, was 57.18 [36.67, 82.39]. Mean [95% CI]. MSE = 1.43e-04 s^2^.

Mass (kg)	Observed Duration (s)	Net Reward Rate Prediction (s)
0	0.780 [0.730, 0.831]	0.783 [0.743, 0.829]
1.36	0.843 [0.781, 0.906]	0.838 [0.794, 0.888]
2.27	0.867 [0.798, 0.936]	0.865 [0.820, 0.918]
3.64	0.904 [0.833, 0.975]	0.900 [0.853, 0.955]

In summary, we found that mass increased the metabolic cost of reaching and led to slower preferred movement speeds. These preferred movement durations could not be fully explained by gross metabolic cost minimization alone but were best explained as the outcome of an implicit decision aimed at maximizing the net reward rate of the movement, where net reward reflects the total reward to be acquired, the probability of acquiring that reward, and the metabolic cost of the movement.

### Determining the combined effects of mass and accuracy on preferred movement duration

Our results thus far demonstrate that increasing metabolic cost exerts a substantial influence on the selection movement speed. What remains unclear however is how effort and accuracy interact to determine movement speed. Maximization of net reward rate assumes that effort and accuracy costs sum linearly to exert their combined influence on movement duration. However, it is possible that the effect of effort on duration may be mitigated (or augmented) when greater accuracy is demanded. To investigate this, we ran an additional experiment, experiment 3, in which the accuracy requirements of the movement were increased by changing the success criterion for completing the movement. We reduced the target to a thin arc of 7 degrees with a thickness of 1 cm within which subjects were required to stop the cursor (r = 0.4 cm) (Fig 1B); this experiment represented a more stringent criterion than experiment 2. Thus, we titrated the effect of accuracy on movement speed while keeping the modulation of added mass the same.

As expected, there was an effect of an experiment’s accuracy requirements on the preferred movement duration (ANOVA, p = 8.85e-5) and peak velocity (ANOVA, p < 1.17e-7). Experiment 3, with its smaller target, yielded longer movement durations and slower peak velocities (Fig 3C and 3E). Yet even with the increased constraint on accuracy, movements slowed with added mass. Interestingly, the changes in movement duration due to mass were similar in extent to that observed in experiment 2 (exp 3: *β* = 0.034 ± 0.001; exp 2: *β* = 0.032 ± 0.001; all p < 2e-16) (Fig 3D). Additionally, added mass influenced peak velocity similarly across both experiments (exp 3: *β* = -0.011 ± 0.0002; exp 2: *β* = -0.013 ± 0.0002; all, p < 2e-16) (Fig 3F). Thus, the effect of mass on movement duration was conserved across accuracy constraints.

We next returned to the net reward rate model defined in Eq 5 to investigate whether it could explain the combined effects of accuracy and effort on movement duration. The speed-accuracy tradeoff defined in Eq 6 was first re-calculated using accuracy constraints that reflected those in experiment 3. Taking endpoint accuracy from experiment 1, we redefined a successful/accurate reach as one that instead finished within a 1 cm wide arc of 7 degrees (~2.44 cm arc length). This resulted in a new set of parameters for the speed-accuracy tradeoff (*β*_0_ = -2.81 ± 0.16; *β*_1_ = 6.06 ± 0.13; *β*_2_ = -0.09 ± 0.01; all p < 2e-16). Using these new accuracy parameters and the same bootstrapped reward values fit from experiment 2 (α = 57.18 [36.67, 82.39]), we once again used the net reward rate model to predict selected movement durations (Table 4).

**Table 4:** Experiment 3 observed durations compared to those predicted by maximizing net reward rate. The fitted free parameter α (57.182 [36.669, 82.390]) from experiment 2 was used. Mean [95% CI]. MSE = 1.82e-03.

Mass (kg)	Observed Duration (s)	Net Reward Rate Prediction (s)
0	0.846 [0.790, 0.901]	0.884 [0.861, 0.912]
1.36	0.902 [0.837, 0.967]	0.929 [0.903, 0.962]
2.27	0.927 [0.864, 0.989]	0.954 [0.925, 0.988]
3.64	0.973 [0.902, 1.045]	0.986 [0.956, 1.022]

Remarkably, we found that, without fitting any parameters to the observed movement durations in experiment 3, the net reward rate model nonetheless captured the data very well (MSE = 1.82e-03 s^2^) (Fig 3B), with no significant differences between the data and model predictions.

While these findings indicated that accuracy and metabolic cost each made substantial, yet independent, contributions to movement speed, the possibility that the increased mass led to either real or perceived reductions in accuracy remained. To address this, we conducted a fourth and final experiment, where we relaxed the criterion for movement success. Subjects were only required to move the cursor through the quadrant and reach back to the home circle, and visual feedback of the cursor was removed (Fig 1B). The purpose of this last experiment was to determine the effect of effort in the absence of any accuracy costs, to the extent possible. Importantly, we did not seek to compare movement durations to the previous experiments, as the movements themselves were different (out-and-stop vs. out-and-back). Our focus was on the effect of mass on within-experiment changes in movement speed. With accuracy costs removed, movement durations still slowed with increasing effort and the extent of change was similar to those observed in experiments 2 and 3 (exp 4: *β* = 0.038 ± 0.001; exp 3: *β* = 0.034 ± 0.001; exp 2: *β* = 0.032 ± 0.001; all p < 2e-16) (Fig 3D). This is more clearly shown when comparing the normalized changes in movement duration with added mass, relative to the 0 kg mass condition across experiments (Table 5). Added mass also slowed peak velocity of the reaching movements to the same extent across all three experiments (exp 4: *β* = -0.020 ± 0.0003; exp 3: *β* = -0.011 ± 0.0002; exp 2: *β* = -0.013 ± 0.0002; all p < 2e-16) (Fig 3F). Even when accuracy costs were negligible, mass led to a significant increase in movement duration and reduction in speed. This suggests that the changes in movement duration are driven by mass-related increases in effort, and not driven by mass-related changes in accuracy.

**Table 5:** Normalized movement durations for experiments 2, 3, and 4, and predicted by gross metabolic minimization. Normalized values determined via dividing by mean duration of 0 kg condition. Mean ± SE.

Mass (kg)	Exp. 2	Exp. 3	Exp. 4	Met. Prediction
0	1 ± 0.052	1 ± 0.045	1 ± 0.064	1 ± 0.021
1.36	1.085 ± 0.062	1.067 ± 0.043	1.124 ± 0.086	1.067 ± 0.019
2.27	1.112 ± 0.054	1.096 ± 0.047	1.175 ± 0.081	1.100 ± 0.020
3.64	1.162 ± 0.06	1.15 ± 0.042	1.234 ± 0.083	1.140 ± 0.020

In summary, these three experiments reveal that the qualitative relationship between metabolic cost and movement duration is preserved across accuracy conditions. This indicates that both the accuracy and metabolic cost of a movement significantly and independently modulate the selection of movement duration.

### The role of temporal discounting

Net reward rate assumes temporal discounting of both the reward to be acquired and effort to be expended. Is a time cost critical for explaining the observed movement durations? To investigate, we tested two additional models. First, we considered a movement utility with no time costs (Eq 8), which only incorporated accuracy and effort. We also considered another movement utility, that only imposed a time cost on reward, and not effort (Eq 9).


$$J_{no\;time\;cost}=\alpha P(\alpha |m,t_{m})-e_{r}-e_{m}$$ (8)

$$J_{reward\;time\;cost}=\frac{\alpha P(\alpha |m,t_{m})}{t_{r}+t_{m}}-e_{r}-e_{m}$$ (9)

**Fig 4:**
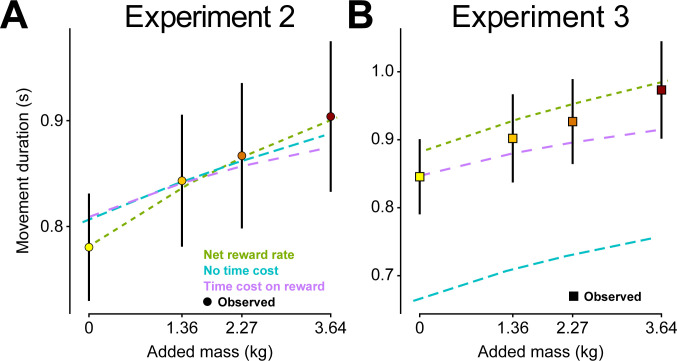
The role of temporal discounting on model predictions. (A) Model fits to average movement durations in experiment 2 when using alternative forms of temporal discounting. (B) Movement duration predictions to experiment 3 data, using the same *α* alpha parameter fit to experiment 2 for each form of utility. Observed data from experiments 2 and 3 shown with vertical bars representing 95% confidence intervals.

Fig 4 presents the fits of all three forms of movement utility to the average data from experiments 2 and 3. Both a utility with no time cost (Eq 8, α = 259.21) and one with only a time cost on reward (Eq 9, α = -94.22) were able to capture movement durations in experiment 2, although with greater error than our original net reward rate formulation (Fig 4A and Table 6). In trying to predict movement durations in experiment 3, a clearer advantage for temporal discounting emerged (Fig 4B). Specifically, we could not explain movement durations in experiment 3 with a utility that did not consider any temporal discounting. However, a cost of time on reward alone fared nearly as well as net reward rate. Thus, our findings build upon prior work [7,9,15,23,28–32]and further support a role for temporal discounting on the choice of movement duration.

**Table 6:** MSE (s^2^) from movement duration predictions when fitting to average experiment 2 data using alternative forms of temporal discounting in movement utility. MSE for experiment 3 predictions used the same α parameter as fit to experiment 2 for each form of utility, respectively.

	MSE Exp. 2	MSE Exp. 3
**Net reward rate (Eq 5)**	9.58e-06	8.53e-04
**No time cost (Eq 8)**	2.41e-04	3.94e-02
**Time cost on reward (Eq 9)**	4.49e-04	1.20e-03

### Effort increased reaction time

**Fig 5:**
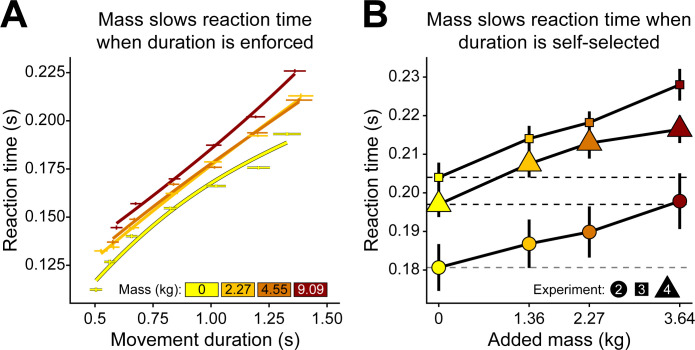
Reaction time slows with added mass. (A) Experiment 1 reaction times. At any given enforced movement duration, reaction times increased with added mass. (B) Experiments 2–4 reaction times. When movement durations were freely self-selected, reaction times increased with added mass across all accuracy conditions. The quickest reaction times were seen in experiment 2 (medium accuracy), then followed by experiment 4 (no accuracy), and lastly by experiment 3 (high accuracy).

An interesting finding across all experiments was that the reaction time chosen by the subject was also significantly modulated by the added mass condition. In experiment 1, while movement speed was restricted, reaction time was still freely chosen. Added mass led to an increase in these self-selected reaction times (*β* = 2.40e-3 ± 1.15e-4, p < 2e-16) (Fig 5A). In experiments 2, 3, and 4 when movement speed was also self-selected, we found that this relation between reaction time and added mass was preserved (*β* = 5.61e-3 ± 2.56e-4, p < 2e-16) (Fig 5B).

## Discussion

The goal of this study was to understand how effort, represented as gross metabolic cost, affects reach speed across a range of accuracy constraints. We found that adding mass at the hand increased the metabolic cost of reaching and led subjects to make slower movements. When comparing the speeds that minimized gross metabolic costs to the empirical preferred reach duration of subjects, we found that minimizing metabolic cost well-predicted subjects’ slowing of movement with added mass. However, individuals still moved more slowly, in an absolute sense, than predicted by minimizing gross metabolic cost alone. Incorporating an accuracy cost more effectively explained preferred movement durations. Altogether, these results suggest that effort in form of metabolic cost is accounted for when determining the speed of reaching movements, but that other factors, namely accuracy and time, are incorporated in tandem.

### Movement duration maximizes net reward rate

A novel aspect of our work is demonstrating that a normative model of movement decision making can explain the choice of movement speed when movements become more effortful and when greater accuracy is required. Others have attempted to use optimal control models to understand changes in movement control with changing accuracy requirements and movement distances [13,24–26]. While our approach does not describe the trajectory of the movement as it unfolds, we instead focus on explicitly varying effort via metabolic cost and accuracy, and comparing model predictions directly to experimental data. The ability to account for movement durations across a range of effort and accuracy requirements was dependent upon a number of key aspects of the net reward rate utility.

First, net reward rate represents effort as metabolic cost. A central question in motor control research is the definition of movement effort. We approach this question from the perspective of locomotion energetics [33,34] and behavioral ecology [35,36], where effort is quantified as the metabolic energy expended. In contrast, traditional optimal control models of reaching movements often represent movement effort as the integral of squared forces or motor commands ([24,37,38], but see 13). By measuring metabolic cost as a function of mass, we show that metabolic costs scale linearly with added mass. This is distinct from integral of squared force, which would scale quadratically with mass in these movements. Further, we show that the change in movement speeds with added mass is explained well by metabolic cost minimization. In contrast, squared force, by virtue of its quadratic scaling with mass, would make dramatically different predictions for the influence of mass on movement duration. Beyond the trivial explanation that there is no non-zero optimal duration if reaching effort is represented as squared force, even incorporating squared force in the net reward rate equation would predict a different relation between mass and optimal duration. Thus, our findings point to a representation of effort as metabolic cost, rather than sum of squared forces, in the implicit control of movement duration.

To account for accuracy, we incorporated the probability of reward based on movement speed. This addition reflects the speed accuracy trade-off, where faster movements tend to become less accurate [18]. The opposite is also true: when accuracy requirements become more strict movements slow down to maintain performance. Previous work has shown that depending on reach distance, variability of the movement changes [39]. However, this study did not investigate how movement variability affects self-selected speeds, only the extent of movement excursion. Work by Peternel et al. [24] uses an optimal control model to qualitatively capture slowing of movement with accuracy constraints, but the model predicts significantly faster movements than those observed experimentally. By including a probability of reward that is dependent on movement speed and mass into utility, in addition to metabolic cost and time, we are able to reasonably predict the self-selected movement speeds across two experiments with a single α parameter.

To complete a movement, a short planning phase exists during the reaction time period prior to movement initiation. To quantify the total cost of a movement more accurately, we need to account for this cost of reacting, or waiting. The added term $\dot{e_{r}}t_{r}$ accounts for the energy used while subjects are in the chair, planning the movement, but not yet moving. Adding this term aids the utility prediction by accounting for the effort expended during this planning phase, proportional to their resting metabolic rate. Recent work has investigated the determination of both an optimal reaction time and movement time that maximizes this utility [31].

Across multiple studies, it has been shown that the passage of time carries an inherent cost thereby preventing us from moving slower than we do [23,29]. This cost of time has been more explicitly incorporated into the utility of a movement in different studies [28–30]. In our model, both reward and effort are divided by time, or in other words, net reward is discounted by time. We found that a movement utility that only enforced a time cost on reward was able to capture changes in movement duration across the two experiments, albeit to a slightly lesser extent than net reward rate. Importantly, a movement utility without any time cost performed markedly worse. Taken together, our results provide further support for the existence of a time cost on movement and suggest that both effort and reward are temporally discounted. However, a definitive conclusion on the specific form of temporal costs requires further study.

### Mass increases metabolic cost of reaching

Here we present the first measures of the effect of mass on the metabolic cost of reaching. We show that similar to walking [20], mass increases the metabolic cost of reaching in a nearly linear manner. However, they found that supporting the load during standing also increased metabolic cost. We do not observe this, possibly because participants’ arms were supported against gravity and thus the resting rate is unaffected by mass. This is supported by the fact that the coefficient on mass (*k*) in our alternative metabolic model (Eq 19 in Methods), was not significantly different from zero. This difference between protocols may also explain why, in contrast to walking, in reaching we observe a shift in the metabolically optimal movement speed with added mass.

### Metabolic minima and slowing of movement

One common method used to explain movement speed is to minimize the total metabolic cost of the movement. Minimizing metabolic cost of transport has been used to explain preferred walking and running velocity [33,40,41]. While a convenient measure, cost of transport may not fully explain observed locomotion slowing in humans when carrying greater loads [21], as the metabolically optimal speed does not always shift with increasing mass [20]. In our study, we found that the metabolic minimum slows with increasing mass, but that preferred durations were slower than predicted by these gross metabolic minima.

Interestingly, we saw that the *change* in the metabolically optimal duration is also very similar to the change in self-selected movement durations. According to our metabolic model, the movement durations predicted by minimizing gross metabolic cost increased by 6.7%, 10.0%, and 14.0% with the masses seen in experiment 2 as compared to 0 kg (Table 5). Empirical preferred durations from experiment 2 and 3 show similar increases. While metabolic cost may not alone predict movement speed in absolute units, it still was an important factor in determining its changes.

### Accuracy

We predicted the discrepancies between metabolic minimum predictions and empirical preferred movement durations may have been due to an additional cost of accuracy. The speed-accuracy tradeoff predicts that smaller targets should incur a reduction in movement speed to sustain performance and, additionally, that slower movements are more accurate than fast movements when moving toward a target of the same size [18,42]. We found that, indeed, slower movement durations increased reach accuracy across all experiments and when the accuracy requirements were altered, preferred reach durations adjusted accordingly (Fig 3).

We also found evidence for an effect of mass on accuracy. In experiment 1 there was an effect of mass on endpoint error (i.e., accuracy) such that heavier loads worsened (shifted to the right) the speed-accuracy curve primarily at the faster speeds. However, during experiments 2 and 3, the self-selected speeds were slow enough for mass to have a negligible effect on accuracy.

### Reaction time

Our results show that imminent effort affects the reaction time of the movement. Across all our experiments, reaction time was self-selected by the subjects (including experiment 1) but consistently slowed with the prospect of added mass. This result mirrors earlier work on upper-limb movements showing that an increase in required distance [43,44] or isometric force production [45,46] leads to a lengthening of reaction time or latency of the action. While the slowing of reaction time has generally been explained as a consequence of increased evidence integration, usually due to increased uncertainty [47,48], it is possible that a general decision variable that tracks utility of a movement is being integrated over this duration. Therefore, a higher effort, lower utility movement that takes longer to integrate a decision variable to a threshold (by either reducing the rate of evidence accrual or by changing the threshold itself) could explain the slowing of reaction time [2].

An alternative theory postulates that the speed and latency of actions is inherently affected by the opportunity cost of time [7,49], which is proportional to the rate of reward/utility of the environment in which the movements are being made. In other words, the utility of a movement is not just determined by immediate factors but also by the past experiences and future expectations of an environment. A more effortful environment has a lower opportunity cost of time, owing to lower net utility rate, and thereby promotes more slothful reaction and movement times. Further evidence of this is seen in experiment 1 wherein subjects react slowly not just to added mass but also to increases in enforced movement duration (Fig 5A).

The duration between stimulus onset and initiation of a movement encompasses cognitive, neural and physiological processes that result in a movement being successfully initiated and completed. From a neurophysiological standpoint, an increase in required effort could increase the duration required to prepare or “organize” the movements [50] and generate the appropriate force [51]. Further research is required to disambiguate the various possible effects of added effort on reaction time.

### Limitations and future work

While our model of movement speed was able to adequately adjust across contexts, there were still many limitations introduced during its construction. First, we estimated speed-accuracy tradeoff curves from experiment 1, which was a separate task with somewhat differing goals. While accuracy was required and encouraged in experiment 1, accuracy itself did not carry any penalty to task performance. In other words, missing the target in experiment 1 did not result in a loss of reward, points, or failure. Thus, it is possible that generalizing experiment 1 endpoint errors to estimate accuracy in experiments 2 and 3 likely introduced error. Along this line, we opted to exclude modeling of experiment 4 due to its out-and-back nature differing from the out-and-stop paradigm of the other experiments.

Second, the “reward” in our experiments was of nebulous value and was fit as a free parameter in our model. We opted to increase model generalizability by fitting reward value (α) from experiment 2 data, and then using the same α in predicting experiment 3. Regardless, it is possible that some of the incongruities between the metabolically predicted preferred durations and the empirical durations could have been due to inadequately appraising the reward, as humans and nonhuman primates accelerate movement speeds when expecting rewards and following a history of rewards [1,8,9]. Individuals opted to move more slowly than predicted by minimizing effort alone and we assumed this was due to the cost of accuracy; however, it is also possible that the value assigned to these movements was lower than predicted.

Third, the model presented here does not differentiate between the objective, metabolic effort of movement and the subjective perception of this effort. During our paradigms, we used a constant set of masses across all individuals, rather than scaling the applied mass based on body mass or strength. Participants may have perceived the effort of moving, for example, the 2.27 kg mass differently dependent upon strength, dopamine levels, or even fatigue [52–54]. Again, observed differences in predicted and actual movement durations could also be the result of individual differences in effort sensitivity.

Lastly, our reward rate model accounts for both reaction and movement times. We used measured reaction times from experiments 2 and 3, and only solved for movement time. Future iterations of this model should seek to optimize for both reaction and movement times. Successfully modeling reaction times may provide valuable information regarding how utility of a movement impacts this planning phase and may offer an additional parameter that an individual can tune when adjusting their strategy [31,55].

## Conclusions

In this study we examined the interaction of metabolic cost and accuracy on the speed, or vigor, of reaching movements. Added mass contributed to an increase in metabolic cost, which subsequently led to slower self-selected movement speeds. While exclusively minimizing the metabolic cost of a movement was remarkably capable of explaining changes in speed with added mass, it was not able to explain absolute preferred durations. Instead, using a neuroeconomic framework that incorporated accuracy and time, alongside effort, we were able to reasonably predict the observed movement durations within distinct environments. Collectively, our results provide novel evidence for the joint importance of accuracy, time, and effort represented as metabolic cost on reaching speed. Our work here lays fundamental knowledge regarding the metabolic costs of reaching movements and sets the stage for understanding the contributions of effort and accuracy to altered movements across a range of movement disorders.

## Methods

### Ethics statement

The study was approved by the University of Colorado Boulder Institutional Review Board. All subjects provided their written informed consent before they participated in the study.

This study is composed of four experiments and a model-based approach to explain preferred movement duration. The first experiment measured the effect of mass and speed on the metabolic power of reaching. The remaining three experiments determined how mass and accuracy coalesce to influence preferred reaching speed. Our modeling approach used the measured metabolic data from experiment 1 to determine which movement currency can best explain the preferred movement durations observed in experiments 2 and 3.

### Experiment 1 –Effect of mass on metabolic power

Eight right-handed subjects (5 male; 28.9 ± 5.5 years; 66.7 ± 11.7 kg body mass; 173.4 ± 10.4 cm height) completed the experiment 1. All subjects except one completed the experiment in two sessions. The remaining subject completed the protocol over 3 sessions. All participants reported no neurological, cardiovascular, or biomechanical problems. Subjects gave written informed consent, as approved by the University of Colorado Institutional Review Board.

#### Protocol

Subjects completed reaching movements with varying speed and mass requirements. Reach kinematic and metabolic data was collected as a function of mass and speed (Fig 1A). Subjects sat in a chair that was height adjusted to place the screen ~3 feet in front and 1 foot above of the subjects’ line of vision, with their arm in a horizontal planar position. They were trained to move a cursor from a home circle and stop at a target circle within a specified time window. Subjects made reaching movements in seven distinct time windows across four different masses (Fig 1A). A block refers to one speed combined with one mass condition. The number of trials per block was determined such that each block consisted of five minutes of reaching at the desired speed, where the first 20 trials of each block were used for training. To begin a trial subjects held a circular cursor (r = 0.4 cm, yellow colored) within the home circle (r = 1.1 cm, white circle) location for 200 ms. The home circle then disappeared and a target circle 10 cm away (r = 1.4 cm) appeared randomly at 45, 135, 225, or 315 degrees from the right horizontal. In training, a blue dot would make a simulated movement from the home circle to the target circle using a minimum jerk trajectory. Feedback on movement duration was given when the center of the cursor was within the target the first time. If subjects moved too slow the target circle would turn grey, whereas if the subject moved too quickly the target would turn green. Appropriately timed movements resulted in the target flashing yellow and a pleasant tone. Upon completing an outward reaching trial, the home and target circle would swap locations and the subject would make another reaching movement towards the center of the screen. Subjects completed four different mass conditions and six different speeds. The completed mass conditions were 0 kg, 2.3 kg, 4.5 kg, and 9.1 kg of added mass at the robot handle which supported the vertical mass. The seven different time windows were: Very, Very Slow (VVS, 1.25–1.35 s, 160 trials), Very Slow (VS, 1.05–1.15, 170 trials), Slow (S, 0.85–0.95 s, 200 trials), Medium (M, 0.65–0.75 s, 220 trials), Fast (F, 0.45–0.55 s, 240 trials), Very Fast (VF, 0.325–0.425 s, 250 trials), and Very, Very Fast (VVF, 0.225–0.275 s, 260 trials). For 0 kg and 2.3 kg added, subjects would complete the faster speed conditions of VS to VVF. For 4.5 kg and 9.1 kg added, subjects would complete the slower speed conditions of VVS to VF.

#### Metabolic data collection

Metabolic data was collected for the duration of each five-minute reaching block. Subjects wore a nose clip and breathed into a mouthpiece, connected to a metabolic cart (ParvoMedics, TrueOne 2400), which measured $\dot{VO_{2}}$ consumption and $\dot{VCO_{2}}$ production. Subjects were required to be well rested and have fasted for 8 hours before testing. Testing sessions began with the subject resting in a seated position in a chair for 10 minutes. Three baseline readings were then taken for 5 minutes each before the experimental protocol began. During these baseline readings, subjects sat quietly in the experiment chair, and held the robotic arm manipulandum. Subjects then began the arm reaching trials. Five-minute rest periods were provided between each block of reaching trials.

### Experiments 2, 3, & 4—Effect of mass on preferred movement duration

Experiments 2–4 measured the effect of mass on preferred movement duration with different accuracy constraints. In contrast to experiment 1, movement duration was not constrained in these experiments. Rather, subjects were allowed to make self-paced movements. In each experiment, a separate cohort made seated horizontal arm reaching movements using a robotic arm manipulandum (Interactive Motion Technologies Shoulder-Elbow Robot 2) positioned similarly to experiment 1 (Fig 1A). All subjects reported no neurological, cardiovascular, or biomechanical problems. Subjects provided written informed consent, as approved by the University of Colorado Institutional Review Board.

#### Kinematic data collection

Across all three experiments, subjects made reaching movements for five blocks of mass conditions. The five blocks were a familiarization block, 0 kg, 1.36 kg, 2.27 kg, and 3.64 kg added at the hand. The order of the weighted conditions was randomized for each subject. The downward weight of the added masses was supported by the robot, so these masses only added inertial effects to the arm. To begin a trial subjects held a circular cursor (r = 0.4 cm, yellow colored) within the home circle (r = 1.1 cm, white circle) location for 200 ms. The home circle then disappeared and a target (shape dependent on experiment) 10 cm away appeared pseudo randomly at 45, 135, 225, or 315 degrees from the right horizontal (Fig 1B). Reaches in experiments 2–4 were self-paced with no imposed time restrictions.

*Experiment 2*: In experiment 2, subjects reached to a target the same size as that provided in experiment 1. Twelve right-handed subjects (8 male; 26.2 ± 3.1 years; 68.4 kg ± 4.4 kg body mass; 173.6 cm ± 11.1 cm height) completed the protocol (Fig 1B). Subjects underwent 5 different blocks of 400 reaching movements (200 out and back movements) to four different targets. The subjects would make horizontal arm reaching movements towards a circular target, identical to experiment 1 (r = 1.4 cm, red color). To ensure subjects came to a complete stop before the trial ended, the target dot would explode after the cursor remained in the target for 300 ms and the velocity during that time was under 0.5 mm/s.

To ensure that the effects of mass on movement speed were not influenced by the size of the target or accuracy costs, we ran experiments 3 and 4 in which we altered the shape of the target to change the accuracy requirements:

*Experiment 3*: The purpose of experiment 3 was to determine the effect of a tighter accuracy constraint (and mass) on movement duration. Thus, this experiment had tighter accuracy constraints in the radial direction (1 cm) compared to experiment 2 (2.8 cm). Twelve subjects (9 male; 25.0 ± 3.61 years; 67.5 kg ± 2.99 kg weight; 171.5 ± 7.58 cm height) completed the protocol. Experiment 3 was identical to experiment 2, except that the targets were smaller and different in shape. Subjects needed to stop between 10 cm and 11 cm from the home circle along a 7 degree (~2.44 cm arc length) arc (Fig 1B). If successful, the target arc turned green. If subjects overshot the target (went past 11 cm) the target would turn red indicating an overshoot.

*Experiment 4*: The purpose of experiment 4 was to determine the effect of mass on preferred movement speed without any accuracy constraints by using a very large target. Experiment 4 was completed by eighteen subjects (9 male; 25.1 ± 3.7 years). Subjects completed 100 out and back movements for familiarization, and 200 out and back movements for each mass condition. The “target” was a 90-degree section of a circle that subjects had to reach towards. However, subjects did not need to stop at any specific location, just cross the target arc, turn around, and return to the home circle. In this experiment we used the point that they turned around (i.e., maximum excursion) as the end of their movement.

### Data acquisition and analysis

For all experiments [1–4], robot handle X (mediolateral) and Y (anteroposterior) position data was recorded at 200 Hz and processed in MATLAB 2019a. Position data was filtered using a fourth order low-pass Butterworth filter (cutoff frequency 10 Hz) and differentiated using a double five-point differentiation to obtain velocity and acceleration. To calculate radial velocity, we calculated the Euclidean distance from the home circle and differentiated using five-point differentiation.

#### Metabolic processing

In experiment 1, we measured gross metabolic rate. Gross metabolic rate was calculated in joules per second, $\dot{e}$, using the method described by Brockway (Eq 10) (Brockway, 1987):

$$\dot{e}=16.58\dot{V}O_{2}+4.51\dot{V}CO_{2}$$ (10)

Average baseline metabolic rate from the three baseline sessions was subtracted from gross metabolic rate to determine the metabolic rate associated with the reaching movement only, or net metabolic rate. Metabolic rate was calculated using the final 3 minutes of each block to ensure steady state. Realized movement durations were similarly calculated using the final 3 minutes within a block. The raw metabolic rate measured by the metabolic cart (${\dot{e}}_{parvo}$) was then normalized by the fraction of time spent moving to calculate gross metabolic rate (W) and gross metabolic cost (J) or the reaching movement:

$$T_{m}=\sum t_{m}$$ (11)

$$T_{trial}=T_{m}+\sum \left(t_{i}+t_{r}\right)$$ (12)

$${\dot{e}}_{m}=\frac{{\dot{e}}_{parvo}{(T}_{trial})-{\dot{e}}_{r}(T_{trial}-T_{m})}{T_{m}}$$ (13)

$$e_{m}=\frac{\dot{e_{m}}(T_{m})}{n}$$ (14)

Where *T*_*m*_ is the sum of all movement times (*t*_*m*_),*T*_*trial*_ is the sum of all total trial times which accounts for the time spent not moving during the reaction time (*t*_*r*_) and intertrial periods (*t_i_*), ${\dot{e}}_{parvo}$ is the “total” metabolic rate being measured by the Parvo metabolic cart, $\dot{e_{r}}$ is the measured resting rate, and ${\dot{e}}_{m}$ is thus the gross metabolic rate when moving only. Lastly, we calculate gross cost per movement (*e*_*m*_) by multiplying ${\dot{e}}_{m}$ by the average movement duration (sum of all movement times divided by the number of trials, *n*) (Eq 14).

#### Movement onset, offset, endpoint, and accuracy

Many of our metrics are dependent on identifying the instant the movement began (movement onset) and the instant the movement ended (movement offset). Movement onset algorithms are often influenced by movement speed, and we wanted to minimize this effect [44,56]. Thus, to detect movement onset and offset, we used a custom algorithm for all experiments that took into account both velocity and acceleration toward the target. It detected movement onset when velocity and acceleration standard deviation in a moving window were above pre-determined thresholds (0.0006 m/s and 0.0075 m/s^2^, respectively). The pseudocode can be found in our online repository. Our algorithm led to detecting movement onset much earlier than typical velocity thresholding algorithms. Additionally, we validated our onset detection method against other algorithms used in the literature [57], and the effect of mass on both reaction time and movement time results were agnostic to the method used.

Movement endpoints were used to detect whether a movement was accurate or not when fitting speed-accuracy curves. Endpoint was calculated at movement offset for each experiment, respectively, and was the two-dimensional Cartesian coordinate for the center of the cursor. With the known coordinates and size of the targets, we could calculate the probability of reach success. A reach was deemed successful if the endpoint landed within the target.

In addition to the probability of reach success, we calculated more continuous measures of movement accuracy. Total error was calculated as the Euclidean distance between the movement endpoint and target center. We also calculated the variance of the angular and radial endpoint coordinates. In experiment 1, all accuracy metrics (probability of success, total error, angular and radial variance) varied strongly with movement duration with an additional small influence of mass. We chose to report and move forward with probability of success as our main measure of accuracy because the net reward rate calculation is based on the probability of successfully reaching the target. Hence this measure is critical. Secondly, probability of success essentially encompasses the continuous measures of accuracy, but in the context most relevant for our analysis.

#### Movement duration

Movement duration was calculated as the time between movement onset and movement offset, except for experiment 4 where it was the time between movement onset and maximum excursion.

#### Outlier analysis

In experiment 1, we removed outlier trials from the statistical analysis if they did not complete the movement correctly. Movement kinematic metrics were computed for every trial, then we removed trials based on specific criteria. We removed any trial where the endpoint error was greater than 10 cm (reached the wrong target), the movement duration was less than 0.2 seconds or greater than 2 seconds (did not make the movement), the reaction time was greater than 0.50 s (failed to initiate movement), or the absolute angular error is greater than 50 degrees (reached to wrong target).

For the kinematic statistical analysis, the data were split into the outward and inward reaching components. We only use the outward reaches in the statistics because subjects could fully predict the location of the target (i.e., central home circle) on inward movements, which may affect the kinematics. 27 out of 15925 total outward trials were removed.

In experiments 2, 3, and 4, we removed trials that were outside 1.5x the interquartile range of movement duration, reaction time, reaction velocity, or angular error. Reaches with a maximum excursion of more than 14 cm were also filtered out in experiment 2 and 3. For experiments 2 and 3, statistical and kinematic analyses were again done only on the outward trials. This removed 702 of 9600 outward trials in experiment 2; 671 of 9600 for experiment 3; and 2335 of 14400 trials for experiment 4.

#### Effective mass calculation

For all models, mass was represented by the subject-specific effective mass of the arm, averaged over the four reach directions [15]. Segment lengths and masses are estimated from anthropometric tables.

To determine the effective mass of the arm at a given time point we defined the Jacobian matrix for a two-link model of the arm, Λ, where *l*_1_ is the length of the upper arm and *l*_2_ is the length of the forearm. θ_*s*_ and θ_*e*_ are the shoulder and elbow joint angle respectively:

$$\Lambda =\frac{dx}{d\theta }=[\begin{array}{cc} -l_{1}sin(\theta_{s})-l_{2}sin(\theta_{s}+\theta_{e}) & -l_{2}sin(\theta_{s}+\theta_{e})\\ l_{1}cos(\theta_{s})+l_{2}cos(\theta_{s}+\theta_{e}) & l_{2}cos(\theta_{s}+\theta_{e}) \end{array}]$$ (15)

The inertial matrix (*I*(θ)) is defined in the equation below, where mass is mass added at the hand. The centroid lengths, *r*_1_ and *r*_22_, refer to the centroid length of the upper arm and forearm with mass added. *I*_*COM*,1_ and *I*_*COM*,2_ are the moment of inertia about the center of mass for the upper arm and forearm.


$$I=[\begin{array}{cc} m_{1}r_{1}^{2}+I_{COM,1}+(mass+m_{2})(l_{1}^{2}+r_{22}^{2}+2l_{1}r_{22}cos(\theta_{e}))+I_{COM,2} & (m_{2}+mass)(r_{22}^{2}+l_{1}r_{22}^{2}cos(\theta_{e}))+I_{COM,2}\\ (m_{2}+mass)(r_{22}^{2}+l_{1}r_{22}^{2}cos(\theta_{e}))+I_{COM,2} & m_{2}r_{2}^{2}+mass\cdot l_{2}^{2}+I_{COM,2} \end{array}]$$ (16)

The mass matrix (*M*) is defined as:

$$M={\left(\Lambda^{-1}\right)}^{T}I(\theta )\Lambda^{-1}$$ (17)

We obtain the effective mass, *m*, in a given reach direction by applying a unit vector acceleration in that direction and calculating the magnitude of the resultant force vector. Each subject’s specific effective mass for experiment 1 and 2 was calculated using anthropomorphic measurements and estimates. These are summarized in Table 7, below. The average effective masses in experiment 2 were used for experiment 3.

**Table 7:** Average calculated effective mass (± standard error) for each added mass condition.

Added mass	Effective mass (Exp. 1)	Effective mass (Exps. 2–3)
0 kg	2.44 ± 0.064 kg	2.506 ± 0.073 kg
1.36 kg	--	3.959 ± 0.073 kg
2.27 kg	4.834 ± 0.068 kg	4.894 ± 0.075 kg
3.64 kg	--	6.282 ± 0.076 kg
4.55 kg	7.127 ± 0.070 kg	--
9.09 kg	11.691 ± 0.071 kg	--

### Models of metabolic cost

We used the measured metabolic power data from Experiment 1 to parameterize the metabolic rate of a movement, ${\dot{e}}_{m}$, as a function of mass and movement duration. Parameter estimates were computed using the function *nls* from package *nlstools*. Using the data in experiment 1, we fit gross metabolic power to Eq 18 using average subject effective mass, metabolic power, and movement durations.


$${\dot{e}}_{m}=a+\frac{bm^{i}}{t_{m}^{j}}$$ (18)

The fitted parameters were *a*,*b*,*i*, and *j*, where *a* is an offset for the cost of not moving, *b* a scaling parameter, *i* scales effort with mass, and *j* scales effort with time. Further, *m* represents the effective mass of the movement and *T* represents the movement duration. The best fit parameters (mean ± standard error) were *a* = 98.25 ± 3.05, *b* = 0.86 ± 0.43, *i* = 0.83 ± 0.10, and *j* = 5.83 ± 0.60 (MSE = 656.38 W^2^, AIC = 1750.83).

To compare whether the cost of resting was influenced by added mass, an alternative metabolic cost model was fit to gross metabolic power data that included a term for effective mass that multiplies the *a* parameter (Eq 19). This represents a mass-dependent cost of resting, where *k* is the scaling on the resting mass cost. AIC scores and MSE were calculated for each metabolic cost model.


$${\dot{e}}_{m}=am^{k}+\frac{bm^{i}}{t_{m}^{j}}$$ (19)

In Eq 19, the fitted value for *k* was not statistically different from zero and the model performed similarly compared to Eq 16 (*k* = -0.02 ± 0.04, MSE = 645.68 W^2^, AIC = 1752.63) indicating that the time-invariant component of metabolic power did not change with added mass. Thus, we moved forward with Eq 18.

Lastly, to obtain an expression for the total metabolic cost of the movement, *e*_*m*_, we multiplied ${\dot{e}}_{m}$ by movement duration, *t*_*m*_.


$$e_{m}=at_{m}+\frac{bm^{i}}{t_{m}^{j-1}}$$ (20)

Minimizing Eq 20 provides the metabolically optimal duration for a movement of a given mass. A distribution of the movement times that minimized gross metabolic cost was estimated by bootstrapping. Bootstrap replicates (n = 1000) were randomly resampled from the data with replacement, parameters of Eq 18 were fit, and movement speeds that minimized gross metabolic cost were calculated using Eq 20. We calculated non-parametric bootstrapped 95% confidence intervals of the speeds that minimized cost within each mass condition as the 2.5% and 97.5% quartiles.

### Models of optimal movement duration

Here we describe the modeling analysis employed to calculate predicted optimal movement durations with changing effort and accuracy requirements. We use the group average data for effective mass, accuracy, movement duration, and reaction time.

#### Optimal duration based on maximizing net reward rate

If the goal of the movement is maximizing net reward rate [7,15,31,32], the utility is determined by the sum of the reward of the movement (α) minus the sum of the effort (*E*), both discounted by time (*T*):

$$J=\frac{\alpha -E}{T}$$ (21)

Eq 21 can be expanded further. Total effort (*E*) is broken apart into two parts: first, the metabolic cost of waiting to move, or reacting (*e*_*r*_), which is the resting metabolic rate, $\dot{e_{r}}$, multiplied by the reaction time, *t*_*r*_:

$$e_{r}={\dot{e}}_{r}t_{r}$$ (22)

Second, is again the cost of moving itself, *e*_*m*_, which is parameterized as a function of the movement time, *t*_*m*_, and the mass moved, *m* (Eq 20). Total time (*T*) is likewise split into reaction time, *t*_*r*_, and movement time, *t*_*m*_ components.

The main difference between experiment 2 and 3 was the size of the target, which can be represented as the probability of acquiring reward at a given movement duration, or accuracy costs. To incorporate this speed-accuracy tradeoff into the utility equation (Eq 23), we scale the reward (α) by the probability of stopping within the target given the movement duration and mass, *P*(*α*|*t*_*m*_,*m*):

$$J=\frac{\alpha P(\alpha |t_{m},m)-(e_{r}+e_{m})}{t_{r}+t_{m}}$$ (23)

The probability function, *P*(α|*t*_*m*_,*m*), was determined using target criteria from experiment 2 and 3 and movement kinematic data from experiment 1. Each arm reaching movement in experiment 1 was labeled as a success (= 1) or failure (= 0) according to the success criteria (target size) from experiment 2 and 3. For experiment 2 labeling, we labeled a reach as a success if the endpoint error was less than 1.4 cm. For experiment 3, we labeled each reach as a success if the maximum excursion was less than 11 cm (and greater than 10 cm), and the angular error was less than 7 degrees. After determining if each reach was a success or not, we used a logistic linear mixed effects model (R, *glmer* model) with binomial family and logit link function (Eq 24) and participant as a random effect to fit function *P*(α|*t*_*m*_,*m*) for each experiment (Eq 25) and obtain two sets of beta coefficients.


$$ln(\frac{P}{1-P})=\beta_{0}+\beta_{1}t_{m}+\beta_{2}m$$ (24)

$$P(\alpha |t_{m},m)=\frac{1}{1+e^{-\beta_{0}-\beta_{1}t_{m}-\beta_{2}m}}$$ (25)

For experiment 2, we found the beta coefficients of the regression to be *β*_0_ = -1.20 ± 0.28, *β*_1_ = 5.96 ± 0.19, and *β*_2_ = -0.11 ± 0.01 (all p < 1.5e-05). Experiment 3 had coefficients *β*_0_ = -2.81 ± 0.16, *β*_1_ = 6.06 ± 0.13, and *β*_2_ = -0.09 ± 0.01 (all p < 2e-16).

We now arrive at the full net reward rate utility model:

$$J=\frac{\alpha (\frac{1}{1+e^{-\beta_{0}-\beta_{1}t_{m}-\beta_{2}m}})-(\dot{e_{r}}t_{r}+at_{m}+\frac{bm^{i}}{t_{m}^{j-1}})}{t_{r}+t_{m}}$$ (26)

Reaction times were not solved for but drawn from experiments 2 and 3. The reaction times from experiment 2 were 0.178 ± 0.013, 0.185 ± 0.014, 0.190 ± 0.013, and 0.196 ± 0.013 for 2.47 kg, 3.80 kg, 4.70 kg, 6.10 kg respectively. Experiment 3 reaction times were 0.205 ± 0.013, 0.215 ± 0.013, 0.219 ± 0.013, and 0.229 ± 0.013.

Now, the only parameter left is α, which represents the subjective reward associated with completing the arm reaching movement. We optimized this utility model (Eq 26) by finding the α value (α = 57.182 [36.669, 82.390]) that minimized the sum of squared error between predicted movement durations and the average movement durations from experiment 2. A distribution of α values and movement times that optimized net reward rate was again estimated by bootstrapping. Bootstrap replicates (n = 1000) were randomly resampled from the experiment 2 data with replacement, the α parameter of Eq 26 fit, and movement speeds predicted. We calculated non-parametric bootstrapped 95% confidence intervals of the speeds that optimized net reward rate within each mass condition as the 2.5% and 97.5% quartiles. The same rewards (α) were used in predicting movement durations from experiment 3 to increase generalizability. In other words, no free parameters were fitted directly to the data from experiment 3. Data was analyzed with the function *optimize* in R and a custom written error function.

#### Alternative models of optimal movement duration

To determine the criticality of temporal discounting, we tested two additional models of movement utility, which removed all temporal discounting (Eq 8), or only discounted reward (Eq 9). To compare performance across the three models, we fit each model to the average movement duration data in experiment 2 and predicted average movement durations in experiment 3.

### Statistical tests

Kinematic data was exported to R for statistical analysis. Linear mixed effects models were computed using the *lme4* and *multcomp* package, and the functions used were *lmer*, *glmer*, and *cftest*. To analyze the effect of mass on the kinematic variables we used linear mixed effects models with a random intercept on subject. For experiment 1, we tested fixed effects of added mass and log-transformed movement duration on outcome variables reaction time and log-transformed gross metabolic power. For experiments 2–4 we tested the fixed effects of added mass and movement direction on outcome variables movement duration, peak velocity, and reaction time.

Probability of successfully hitting the target using experiment 1 data and success criteria from experiments 2 and 3 was modeled using a logit-linked binomial generalized linear mixed effects regression model. Success was coded as a binary hit (= 1) or miss (= 0). Subject was a random intercept, while effective mass and movement duration were fixed effects. The intercept (*β*_0_) and slopes on movement duration (*β*_1_) and effective mass (*β*_2_) were the respective coefficients used in Eqs 24 and 25.

The effective mass used in the linear and generalized linear mixed effects models is the respective average effective masses from experiment 1 and experiment 2. Experiment 2 effective mass values were applied to experiment 3.

An ANOVA (*aov* in R) was used to test differences between experimental outcomes (2 and 3). Experimental data was aggregated for each experiment and subject before use in the ANOVA test.
